# Uncommon Combination of Low Alanine Aminotransferase and High Fatty Liver Index Associated With High Charlson Comorbidity Index: Insights From the K7Ps-Study-7

**DOI:** 10.14740/jocmr6525

**Published:** 2026-03-26

**Authors:** Kei Nakajima, Airi Sekine

**Affiliations:** a Department of Nutritional Science, Faculty of Food and Nutritional Sciences, Japan Women’s University, Tokyo 112-8681, Japan; b Saitama Medical Center, Department of Endocrinology and Diabetes, Saitama Medical University, Kawagoe 350-8550, Japan

**Keywords:** Alanine aminotransferase, Charlson Comorbidity Index, Fatty liver, Hepatic enzymes

## Abstract

**Background:**

Elevated serum alanine aminotransferase (ALT) often reflects fatty liver, whereas low ALT has been reported as a predictor of frailty and increased mortality. However, the association between ALT and the Charlson Comorbidity Index (CCI), a measure of comorbidity and long-term mortality, is unknown. Therefore, this study aimed to investigate the association, particularly focusing on the fatty liver index (FLI) and physical characteristics, in a community-based cross-sectional study.

**Methods:**

We examined liver enzymes, CCI, and cardiometabolic factors in 6,418,215 individuals without hepatic cirrhosis. Participants were classified into normal FLI (< 30, n = 4,418,623, NFG) and high FLI group (≥ 30, n = 1,999,592, HFG). A generalized linear model (GLM) was used to assess the association between ALT and CCI.

**Results:**

Overall, all variables including CCI, but except for high-density lipoprotein cholesterol and prevalence of women, were higher in HFG than NFG. Age, CCI, and prevalence of cardiovascular disease and stroke increased with rising ALT levels in NFG, whereas they decreased in HFG (all P < 0.0001, linear regression and Cochran–Armitage trend test). Individuals with lower ALT were older, more often women, and those with lower physical capacity in HFG. Results of GLM demonstrated a U-shaped association between ALT and CCI, with the lowest point at ALT 20–29 U/L, after adjusting for covariates in NFG. By contrast, in HFG, lowest ALT of < 10 U/L (n = 17,261, 0.9%) was most associated with CCI than any other categories of ALT, with the lowest point at ALT of 30–49 U/L. Such trends were not observed in aspartate aminotransferase and γ-glutamyl transferase.

**Conclusions:**

The uncommon combination of low ALT and fatty liver may be associated with high CCI, and older and more often women with the aspects of frailty, which was disclosed by the consideration of fatty liver, suggesting a phenomenon related to obesity paradox.

## Introduction

Early studies suggested that elevated levels of serum alanine aminotransferase (ALT), an enzyme found systemically, but predominantly in the liver, are broadly indicative of fatty liver and metabolic syndrome [1–3]. In recent decades, however, low ALT has been reported as indicative of sarcopenia, frailty, aging, and increased mortality [4–6], all of which interact closely with each other from a pathological point of view [7]. Interestingly, sarcopenia and frailty have likewise been associated with fatty liver and metabolic dysfunction-associated steatotic liver disease (MASLD) [8, 9], although ALT is often elevated in patients with MASLD [2, 3].

Thus, both low and high ALT may be risk factors for various health damages, and the optimal range of serum ALT may vary depending on the disease context [10, 11], reflecting complex and incompletely understood mechanisms.

The Charlson Comorbidity Index (CCI) is a widely used metric for evaluating comorbidity and predicting long-term mortality based on 19 fatal diseases in different clinical populations, including medical, surgical, intensive care unit, trauma, and cancer patients [12, 13]. In the last decade, CCI can be calculated using the data of health checkup and receipt claims in Japan [14, 15]. Accordingly, this cross-sectional study aimed to examine the relationship between serum ALT levels and the CCI, with particular attention to the condition of fatty liver assessed with the fatty liver index (FLI) [16, 17], which likely modifies the relationship, using large data from the National Database of Health Insurance Claims and Specific Health Checkups of Japan (NDB), a comprehensive database encompassing all health insurance users in Japan, including diagnoses and prescriptions.

## Materials and Methods

### Study design

In this epidemiological study, we analyzed annual health checkup data along with disease diagnoses coded according to the International Classification of Diseases, 10th Revision (ICD-10). The study concept and design have been described in detail elsewhere [18]. Since 2008, all Japanese citizens aged 40–74 years have been required to undergo yearly itemized health checkups [19]. The study protocol was approved by the Japanese Ministry of Health, Labor and Welfare (MHLW) in December 2020 (ID No. 0320). In July 2022, we obtained digitally recorded, anonymized data from the MHLW. The study was conducted in accordance with the Declaration of Helsinki and approved by the Institutional Review Board of Japan Women’s University (No. 513, May 11, 2022). The requirement for informed consent was waived because of the retrospective nature of the study and anonymized, hashed data provided by the MHLW as part of its nationwide program for sharing medical data with third parties [20]. This study has examined diagnosed liver disease, particularly chronic hepatitis, cirrhosis, and non-alcoholic steatohepatitis (NASH). The study was registered with the University Hospital Medical Information Network Clinical Trials Registry (UMIN-CTR; ID: UMIN000058227).

### Study population

The study included 10,183,619 adults aged 40–74 years living in the prefectures of the Kanto region who underwent specific health checkups required by the government between April 2018 and March 2019. Kanto area is home to approximately one-third of the entire Japanese population. To protect participant confidentiality, as required by the MHLW, ages were categorized in 5-year intervals (40–44, 45–49, 50–54, 55–59, 60–64, 65–69, and 70–74 years).

Participants with incomplete data and those meeting any of the following criteria were excluded: ALT > 100 U/L, a mild elevation criteria [21]; body mass index (BMI) <15 or ≥ 40 kg/m^2^, which are criteria for extreme anorexia nervosa [22] and severe obesity [23]; aspartate aminotransferase (AST) > 100 U/L [24]; γ-glutamyl transferase (GGT) >100 U/L; glycated hemoglobin (HbA1c) > 10%; triglycerides (TG) > 1,000 mg/dL; or hepatic cirrhosis (International Classification of Diseases, 10th Revision (ICD-10) codes B181, B182, K703, K717, K743, K744, K745, K746, and K761). After applying these criteria, 6,418,215 individuals without liver cirrhosis remained for analysis.

### Laboratory measurements

We retrospectively examined clinical parameters, including BMI, waist circumference (WC), serum ALT, AST, and GGT levels, the CCI, and the FLI [18]. The MHLW has instructed the method of all measurements in the checkup.

The FLI has been used in clinical practice for the past two decades and has demonstrated accuracy in multiple studies [16]. Many studies comparing FLI results with liver biopsy results showed that FLI had a good agreement for diagnosing steatosis (mostly area under the curve (AUC) 0.8 or over) [17].

The participants were classified into five groups based on serum ALT values, following previous studies [4, 5]: low (3–9 U/L), low-normal (10–19 U/L), normal (20–29 U/L), high (30–49 U/L), and very high (50–99 U/L).

Not everyone was screened for fatty liver with abdominal computed tomography (CT), magnetic resonance imaging (MRI), or ultrasonography. We then used FLI, an index integrating obesity- and metabolism-related parameters, based on blood test values (TG and GGT) and central measures (BMI and WC) obtained for all participants.

The FLI was calculated as follows [16, 17]:

$$\text{FLI}=\frac{e^{\left(\text{0.953}\times \text{ln(TG)}+\text{0.139}\times \text{BMI}+\text{0.718}\times \ln\text{(GGT)}+\text{0.053}\times \text{WC}-\text{15.745}\right)}}{\text{1}+e^{\left(\text{0.953}\times \text{ln(TG)}+\text{0.139}\times \text{BMI}+\text{0.718}\times \text{ln(GGT)}+\text{0.053}\times \text{WC}-\text{15.745}\right)}}\times \text{100}$$

Normal and high FLI were defined as < 30 and ≥ 30, respectively [16, 17].

The CCI was calculated based on validated definitions from previous studies [12, 25], with age excluded from the calculation. The detail of methods is described elsewhere [26]. Several CCI components were unavailable in our study, such as hospitalization details, New York Heart Association functional class, dialysis, and post-kidney transplant status. Therefore, lack of these data can lead to incomplete assessments of CCI, although the prevalence of heart failure and renal disease were very small (3.3% and 0.9%, respectively), and participants investigated were physically able to undergo a health checkup.

### Diagnosed liver diseases

Chronic hepatitis was determined if any of the following ICD-10 codes were present in the clinical records: B150, B162, B169, B171, B179, B182, B189, B190, B199, B251, B268, B270, K701, K711, K712, K713, K716, K730, K732, K738, K739, K746, K752, K753, K754, K758, or K759. NASH was identified by ICD-10 code K758.

### Statistical analysis

Data are presented as mean ± standard deviation or median (interquartile range). To estimate average age, age groups were converted into substituted ages (s-ages) corresponding to the median of each group (42, 47, 52, 57, 62, 68, and 72 years, respectively). Trends in continuous and categorical variables among ALT groups were examined using linear regression analysis and the Cochran–Armitage trend test. Comparisons between normal (NFG) and high FLI groups (HFG) were performed with the *t*-test and Chi-square test. Missing values in categorical data were coded as “unavailable” and calculated as usual.

A generalized linear model (GLM) was applied to examine associations between the five ALT groups and continuous variable of CCI, which does not depend on the specific cut-off criteria such as 3 and 4 [12], and evaluates association per 1-point increase, adjusting for covariates (age, sex, clinical factors, AST, GGT, pharmacotherapies for hyper-tension, diabetes, and dyslipidemia, and chronic hepatitis). The statistical significance and contribution of each covariate on CCI were assessed using β coefficient and Wald Chi-square (WCS) values derived from the GLM. As an indicator of significant contribution, standardized β is preferable to β. However, it was hard to standardize all covariates and calculate the standardized β in this study with many categorical covariates. Therefore, we added WCS statistics, another indicator of significant contribution. Acceptable to high correlations are observed among WCS values, standardized β coefficients, and feature importance derived from machine learning in studies with large sample sizes [27]. Large sample size was required to analyze the expected rare cases of lowest ALT group considering many covariates. All analyses were conducted with SAS Enterprise Guide (SAS-EG 7.1) in SAS version 9.4 (SAS Institute, Cary, NC, USA). A two-tailed P value of < 0.05 was considered statistically significant.

## Results

Tables 1 and 2 present the participants’ clinical characteristics according to the five ALT groups and two FLI groups (NFG and HFG). Overall, all variables including s-Age, BMI, TG, HbA1c, and CCI, except for high-density lipoprotein cholesterol, and the prevalence of cardiovascular disease, stroke, hepatic diseases, and NASH, except for women and no regular exercise, were higher in HFG than NFG (*t*-test and Chi-square test, all P < 0.001). This indicates that participants in HFG reflected older population with obesity and cardiometabolic abnormalities. Noteworthy, AST values were significantly lower in HFG when analysis was conducted according to each ALT group except for lowest ALT group (all P < 0.0001).

**Table 1:** Clinical Characteristics of Participants With Normal FLI According to Serum ALT Levels (N = 4,418,623)

ALT (U/L) groups	3–9	10–19	20–29	30–49	50–99
N (% in total)	268,498 (6.1)	2,819,653 (63.8)	1,027,124 (23.3)	269,793 (6.1)	33,555 (0.8)
s-Age	52.1 ± 10.1	55.7 ± 10.3	56 ± 10.0	54.5 ± 9.8	53.6 ± 9.9
Women, n (%)	212,873 (79.3)	1,794,092 (63.6)	452,975 (44.1)	95,569 (35.4)	12,771 (38.1)
Body mass index (kg/m^2^)	20.9 ± 2.3	21.3 ± 2.3	21.8 ± 2.3	22.1 ±2 .3	22.3 ± 2.3
Waist circumference (cm)	75.9 ± 7.0	77.5 ± 7.0	79.0 ± 6.7	79.7 ± 6.5	80 ± 6.4
SBP (mm Hg)	116 ± 16.8	119 ± 17.0	121 ± 16.6	122 ± 16.3	122 ± 16.4
TG (mg/dL)	67 (51–90)	72 (55–97)	77 (58–103)	79 (60–105)	80 (60.9–104)
HDL-C (mg/dL)	69.8 ± 15.5	70.5 ± 16.5	68.3 ± 17.1	65.5 ± 17	62.7 ± 16.5
AST (U/L)	15.7 ± 3.0	19.4 ± 3.8	24.0 ± 4.9	29.6 ± 7.6	42 ± 12.9
ALT (U/L)	8.1 ± 1.1	14.5 ± 2.7	23.2 ± 2.7	35.5 ± 5.0	61.6 ± 11.4
GGT (U/L)	16.0 ± 7.7	21.1 ± 10.8	28.9 ± 15.3	35.4 ± 18.5	40.3 ± 19.7
HbA1c (%)	5.4 ± 0.4	5.5 ± 0.4	5.6 ± 0.5	5.6 ± 0.5	5.7 ± 0.6
CCI	0.5 ± 1.0	0.5 ± 1.0	0.6 ± 1.1	0.6 ± 1.1	0.8 ± 1.2
FLI	5 (3–10)	8 (4–15)	13 (7–21)	17 (10–24)	19 (12–25)
Hepatic diseases, n (%)^a^	205 (0.08)	2156 (0.08)	909 (0.09)	373 (0.14)	103 (0.31)
NASH, n (%)	11 (0.004)	253 (0.01)	277 (0.03)	206 (0.08)	129 (0.38)
Pharmacotherapy^d^					
Hypertension, n (%)	25,778 (9.6)	357,562 (12.7)	151,667 (14.8)	41,431 (15.4)	5,567 (16.6)
Diabetes, n (%)	5,378 (2.0)	70,592 (2.5)	35,727 (3.5)	12,551 (4.7)	2,102 (6.3)
Hyperlipidemia, n (%)	12,647 (4.7)	255,527 (9.1)	138,764 (13.5)	42,068 (15.6)	5,364 (16.0)
History of CVD, n (%)^d^	4,688 (1.8)	62,143 (2.2)	27,718 (2.7)	7,743 (2.9)	957 (2.9)
Unavailable, n (%)^d^	16,265 (6.1)	168,271 (6.0)	63,219 (6.2)	17,097 (6.3)	2,048 (6.1)
History of stroke, n (%)^d^	3,390 (1.3)	36,169 (1.3)	15,042 (1.5)	4,126 (1.5)	524 (1.6)
Unavailable, n (%)^d^	16,284 (6.1)	168,141 (6.0)	63,232 (6.2)	17,108 (6.3)	2,044 (6.1)
Smokers, n (%)^d^	49,284 (18.4)	463,621 (16.4)	182,141 (17.7)	51,223 (19.0)	6,026 (18.0)
Heavy drinkers, n (%)^b, d^	4,456 (1.7)	57,040 (2.0)	27,390 (2.7)	7,911 (2.9)	845 (2.5)
Unavailable, n (%)^d^	70,817 (26.4)	698,311 (24.8)	245,940 (23.9)	66,097 (24.5)	8,962 (26.7)
No regular exercise, n (%)^c, d^	192,419 (71.7)	1761,853 (62.5)	612,925 (59.7)	167,993 (62.3)	22,249 (66.3)
Unavailable, n (%)^d^	23,055 (8.6)	249,813 (8.9)	90,781 (8.849)	23,668 (8.8)	2,806 (8.4)
Slow gait, n (%)^d^	131,571 (49.0)	1177,599 (41.8)	410,529 (40.0)	112,079 (41.5)	14,720 (43.9)
Unavailable, n (%)^d^	26,026 (9.7)	281,543 (10.0)	102,354 (10.0)	26,720 (9.9)	3,184 (9.5)

Data are presented as mean ± standard deviation, median (interquartile range), or n (%). Normal FLI was defined as < 30. ^a^Chronic hepatitis and 27 other conditions as described in the Methods section. ^b^Defined as consuming ≥ 69 g ethanol per drinking session. ^c^Defined as not exercising for ≥ 30 min at least twice per week. ^d^Data are self-reported information. s-Age: substituted age; SBP: systolic blood pressure; TG: triglycerides; HDL-C: high-density lipoprotein cholesterol; ALT: alanine aminotransferase; AST: aspartate aminotransferase; GGT: γ-glutamyl transferase; HbA1c: glycated hemoglobin; CCI: Charlson Comorbidity Index; CVD: cardiovascular disease; NASH: non-alcoholic steatohepatitis; FLI: fatty liver index.

**Table 2:** Clinical Characteristics of Participants With High FLI According to Serum ALT Levels (N = 1,999,592)

ALT (U/L) groups	2–9	10–19	20–29	30–49	50–99
N (% in total)	17,261 (0.9)	551,403 (27.6)	690,221 (34.5)	530,584 (26.5)	210,123 (10.5)
s-Age	58.9 ± 10.5	58.6 ± 10.0	56.6 ± 9.7	54.3 ± 9.4	52.4 ± 9.1
Women, n (%)	8,639 (50.1)	221,916 (40.3)	183,816 (26.6)	103,129 (19.4)	38,892 (18.5)
Body mass index (kg/m^2^)	26.5 ± 3.2	26.2 ± 2.9	26.3 ± 3.0	27.0 ± 3.2	28.2 ± 3.6
Waist circumference (cm)	92.5 ± 7.3	91.4 ± 7.1	91.3 ± 7.3	92.6 ± 8.0	95.3 ± 8.7
SBP (mm Hg)	129.5 ± 16.8	129.4 ± 16.3	129.1 ± 15.9	129.1 ± 15.7	130 ± 15.6
TG (mg/dL)	135 (100–188)	137 (102–187)	140 (104–192)	144 (107–200)	146 (108–203)
HDL-C (mg/dL)	55.6 ± 14.1	56.4 ± 13.6	55 ± 13.2	52.5 ± 12.2	49.8 ± 11
AST (U/L)	16.1 ± 3.8	19.1 ± 3.8	22.7 ± 4.5	28.1 ± 6.4	40.5 ± 11.3
ALT (U/L)	8.1 ± 1.2	15.7 ± 2.5	24.1 ± 2.8	37.2 ± 5.5	65 ± 12.8
GGT (U/L)	28.8 ± 17.4	34.7 ± 18.4	43 ± 20.3	49.6 ± 20.8	56.5 ± 20.1
HbA1c (%)	5.7 ± 0.6	5.7 ± 0.6	5.8 ± 0.6	5.9 ± 0.7	6.0 ± 0.8
CCI	1.1 ± 1.6	0.8 ± 1.3	0.7 ± 1.2	0.7 ± 1.2	0.8 ± 1.2
FLI	42 (35–54)	43 (36–56)	48 (38–63)	56 (43–72)	68 (51–82)
Hepatic diseases, n (%)^a^	24 (0.14)	534 (0.10)	633 (0.09)	513 (0.10)	382 (0.18)
NASH, n (%)	–^d^	201 (0.04)	386 (0.06)	742 (0.14)	933 (0.44)
Pharmacotherapy^e^					
Hypertension, n (%)	6,440 (37.3)	179,251 (32.5)	207,594 (30.1)	156,032 (29.4)	64,268 (30.6)
Diabetes, n (%)	1,882 (10.9)	45,935 (8.3)	53,811 (7.8)	47,414 (8.9)	22,905 (10.9)
Hyperlipidemia, n (%)	3,357 (19.5)	102,933 (18.7)	135,581 (19.6)	109,724 (20.7)	42,882 (20.4)
History of CVD, n (%)^e^	891 (5.2)	23,576 (4.3)	26,810 (3.9)	18,721 (3.5)	6,549 (3.1)
Unavailable, n (%)^e^	1,005 (5.8)	33,517 (6.1)	44,447 (6.4)	35,427 (6.7)	14,205 (6.8)
History of stroke, n (%)^e^	670 (3.9)	13,739 (2.5)	14,222 (2.1)	9,599 (1.8)	3,457 (1.7)
Unavailable, n (%)^e^	998 (5.8)	33,454 (6.1)	44,432 (6.4)	35,425 (6.7)	14,219 (6.8)
Smokers, n (%)^e^	4,884 (28.3)	140,952 (25.6)	183,182 (26.5)	147,547 (27.8)	56,578 (26.9)
Heavy drinkers, n (%)^b, e^	656 (3.8)	24,408 (4.4)	35,061 (5.1)	26,800 (5.1)	9,645 (4.6)
Unavailable, n (%)^e^	4,203 (24.3)	123,277 (22.4)	147,904 (21.4)	119,973 (22.6)	52,844 (25.1)
No regular exercise, n (%)^c, e^	11,393 (66.0)	346,861 (62.9)	442,913 (64.2)	361,026 (68.0)	151,605 (72.2)
Unavailable, n (%)^e^	1,639 (9.5)	53,074 (9.6)	64,758 (9.4)	48,029 (9.1)	19,184 (9.1)
Slow gait, n (%)^e^	9,355 (54.2)	263,270 (47.8)	318,289 (46.1)	253,117 (47.7)	107,283 (51.1)
Unavailable, n (%)^e^	1,907 (11.1)	61,244 (11.1)	74,513 (10.8)	55,161 (10.4)	21,878 (10.4)

Data are presented as mean ± standard deviation, median (interquartile range), or n (%). High FLI were defined as ≥ 30. ^a^Chronic hepatitis and 27 other conditions as described in the Methods section. ^b^Defined as consuming ≥ 69 g ethanol per drinking session. ^c^Defined as not exercising for ≥ 30 min at least twice per week. ^d^Counts of < 10 are not reported, in accordance with guidelines from the Japan Ministry of Health, Labor and Welfare to prevent individual identification. ^e^Data are self-reported information. s-Age: substituted age; SBP: systolic blood pressure; TG: triglycerides; HDL-C: high-density lipoprotein cholesterol; ALT: alanine aminotransferase; AST: aspartate aminotransferase; GGT: γ-glutamyl transferase; HbA1c: glycated hemoglobin; CCI: Charlson Comorbidity Index; CVD: cardiovascular disease; NASH: non-alcoholic steatohepatitis; FLI: fatty liver index.

All continuous variables and the prevalence of pharmacotherapies for hypertension, diabetes, and dyslipidemia, as well as histories of cardiovascular disease and stroke, increased across rising ALT levels in NFG (all P < 0.0001, linear regression, Cochran–Armitage trend test). In contrast, age and CCI decreased across rising ALT levels in HFG, although BMI and WC showed a U-shaped relationship against ALT. The prevalence of pharmacotherapies for hypertension, diabetes, and dyslipidemia, as well as histories of cardiovascular disease and stroke, also decreased with rising ALT levels in HFG (all P < 0.0001).

In addition, the prevalence of no regular exercise and slow gait were greater among participants with lower ALT in both groups (Cochran–Armitage trend test, all P < 0.0001), although they appeared to be a subtle U-shaped relationship. Participants with lowest ALT (n = 17,261, 0.9%) were older and more often women in HFG (Table 2), whereas younger in NFG (Table 1). However, when the participants were restricted to those aged 65 years old or over (n = 1,654,312), such trends in HFG were not found (data not shown).

Table 3 shows the results of the GLM examining associations between ALT groups and continuous CCI. In NFG, very high ALT (50–99 U/L) was more strongly associated with the CCI after adjustment for age and sex than low-normal ALT (10–19 U/L) (β = 0.353, WCS = 4,100) (model 1). However, after additional adjustment for confounders, both low ALT and very high ALT were associated with a higher CCI than normal ALT (20–29 U/L) (β = 0.063, WCS = 766; β = 0.094, WCS = 275), showing a U-shaped association between ALT and the CCI (model 2). The coefficient β in lowest ALT (0.063) was close to that in highest ALT (0.094), although WCS was higher in the former (766) than the latter (275).

**Table 3:** Generalized Linear Model of Categorized ALT for CCI

Categorized ALT	ALT				
	3–9 U/L	10–19 U/L	20–29 U/L	30–49 U/L	50–99 U/L
Participants in NFG (n = 4,418,623)					
Model 1					
β (95% CIs)	0.062 (0.058–0.066)	0 (reference)	0.037 (0.034–0.039)	0.1366 (0.133–0.141)	0.353 (0.342–0.364)
WCS value	930		983	4,484	4,100
Model 2					
β (95% CIs)	0.063 (0.058–0.067)	0.014 (0.012–0.017)	0 (reference)	0.013 (0.009–0.017)	0.094 (0.083–0.105)
WCS value	766	132		37	275
Participants in HFG (n = 1,999,592)					
Model 1					
β (95% CIs)	0.266 (0.248–0.284)	0.011 (0.007–0.015)	0 (reference)	0.089 (0.085–0.093)	0.266 (0.260–0.272)
WCS value	843	26		1,673	7,947
Model 2					
β (95% CIs)	0.251 (0.238–0.267)	0.064 (0.058–0.068)	0.012 (0.008–0.016)	0 (reference)	0.016 (0.010–0.022)
WCS value	858	574	31		25

Normal and high FLI were defined as < 30 and ≥ 30, respectively. All associations were statistically significant (P < 0.0001). Model 1: adjustment for substituted age and sex. Model 2: model 1 plus adjustment for body mass index; systolic blood pressure; high-density lipoprotein cholesterol; triglycerides; HbA1c; pharmacotherapy for hypertension, diabetes, and dyslipidemia; smoking; heavy drinking; no regular exercise; slow gait; history of cardiovascular disease and stroke; hepatic diseases; aspartate aminotransferase; and γ-glutamyl transferase. ALT: alanine aminotransferase; β: coefficient; WCS: Wald Chi-square; FLI: fatty liver index; HbA1c: glycated hemoglobin; CCI: Charlson Comorbidity Index; CI: confidence interval; NFG: normal FLI group; HFG: high FLI group.

In HFG, both lowest ALT and very high ALT were associated with the CCI after adjustment for age and sex (β =0.266, WCS =843; β =0.266, WCS =7,947) (model 1). After further adjustment for covariates, lowest ALT remained most strongly associated with the CCI (β = 0.251, WCS = 858) (model 2). Table 4 shows the results of GLM conducted for other liver enzymes, AST and GGT, for the comparison with ALT. Under model 2 conditions, lowest AST (2–9 U/L) and very high AST (≥ 50 U/L) were also associated with the CCI, regardless of the FLI, with the low-normal range (10–19 U/L) as the reference, although WCS was higher (1,152) in very high AST group. By contrast, very high GGT (≥ 50 U/L) was most significantly associated with the CCI, regardless of the FLI.

**Table 4:** Generalized Linear Model of Categorized AST and GGT for CCI

	2–9 U/L	10–19 U/L	20–29 U/L	30–49 U/L	50–99 U/L
AST					
Participants with normal FLI					
Model 2					
β (95% CIs)	0.553 (0.517–0.588)	0 (reference)	0.022 (0.020–0.024)	0.119 (0.114–0.123)	0.305 (0.289–0.322)
WCS value	933		421	2,604	1,329
Participants with high FLI					
Model 2					
β (95% CIs)	0.952 (0.876–1.028)	0 (reference)	0.018 (0.014–0.022)	0.096 (0.089–0.102)	0.220 (0.208–0.233)
WCS value	602		70	899	1,152
GGT					
Participants with normal FLI					
Model 2					
β (95% CIs)	0.007 (0.00–0.014)	0 (reference)	0.009 (0.007–0.011)	0.022 (0.020–0.025)	0.060 (0.054–0.065)
WCS value	4		68	268	437
Participants with high FLI					
Model 2					
β (95% CIs)	0.077 (0.013 -0.141)	0 (reference)	0.002 (–0.004 to 0.009)	0.014 (0.008–0.020)	0.043 (0.036–0.050)
WCS value	6		1	21	152

Normal and high FLI were defined as < 30 and ≥ 30, respectively. All associations were statistically significant (P < 0.0001). Model 1: adjustment for substituted age and sex. Model 2: model 1 plus adjustment for body mass index; systolic blood pressure; high-density lipoprotein cholesterol; triglycerides; HbA1c; pharmacotherapy for hypertension, diabetes, and dyslipidemia; smoking; heavy drinking; no regular exercise; slow gait; history of cardiovascular disease and stroke; hepatic diseases; ALT; AST; and GGT. ALT: alanine aminotransferase; AST: aspartate aminotransferase; GGT: γ-glutamyl transferase; HbA1c: glycated hemoglobin; CCI: Charlson Comorbidity Index; β: coefficient; WCS: Wald Chi-square; FLI: fatty liver index; CI: confidence interval.

## Discussion

This study demonstrated that lower ALT was positively associated with higher CCI, particularly among participants with a high FLI, suggesting that fatty liver status modifies the clinical meanings of low ALT. This is consistent with previous findings that low ALT predicts frailty, sarcopenia, severe comorbidities and higher mortality [4–6]. In such conditions, fatty liver may play a key role in the background of pathophysiology.

The reference ALT level in the high FLI group was relatively elevated (30–49 U/L), suggesting that the optimal ALT range related to comorbidity and mortality may be higher than previously assumed in individuals with fatty liver [28, 29]. Consistently, participants with lowest ALT in the high FLI group showed a higher prevalence of cardiovascular disease and stroke (5.2% and 3.9%, respectively) in this study, although the possibility of reverse causality cannot be ruled out.

Alternatively, this may be related to the obesity paradox, a phenomenon that patients with many types of cardiovascular diseases have a better prognosis if classified as overweight or obese [30, 31]. Such obese patients are likely to have fatty liver and elevated ALT.

For the last decades, many studies have shown that low ALT level is a risk for cancer, frailty, cardiovascular disease, and chronic obstructive pulmonary disease [4–6]. However, our results demonstrated that low ALT level was not strongly associated with high CCI in participants with normal FLI compared with those with high FLI. Therefore, fatty liver-related metabolic abnormalities and systemic organ impairments may exist in these diseases. Although FLI does not directly measure hepatic fat accumulation and is influenced by obesity-related factors, it remains a practical tool for population-based risk stratification [16, 17].

Our study suggests that low ALT may be associated with a good prognosis when individual is in the condition of less likely to have fatty liver. In contrast, low ALT may be associated with a poor prognosis when individual has fatty liver even with mild grade. These findings may be useful for risk stratification and clinical decision-making in daily practice.

Because fatty liver is often accompanied by elevated ALT, the coexistence of low ALT and fatty liver is uncommon in clinical practice; accordingly, such individuals were rare (0.9%) in the high FLI group. The underlying mechanism of low ALT in the presence of fatty liver remains unclear. Of note, in this study, lowest ALT was unlikely to reflect severely damaged hepatic cells, such as those seen in advanced NASH or chronic hepatitis progressing toward cirrhosis. In general, people with high ALT and fatty liver tend to be younger [32], which is consistent with current results (Table 2). However, individuals with lowest ALT and a high FLI were older, more often women, and more likely to have lower physical capacity (higher prevalence of no regular exercise and slow gait), which may reflect the pathophysiology of sarcopenia and frailty.

The enzyme ALT, particularly the isoform ALT1, is expressed not only in the liver but also in skeletal muscle and kidney [33]. By contrast, the isoform ALT2 is expressed in the heart and skeletal muscle [34]. Therefore, ALT2 might be preferentially reduced in individuals with both low ALT and fatty liver. Further investigation of ALT isoforms and skeletal muscle mass may help clarify the underlying mechanism and the clinical feature linking to low ALT accompanied by fatty liver, although routine clinical assays measure total ALT activity and do not distinguish between these isoforms.

Meanwhile, both lowest and very high AST were also associated with the CCI. However, the reference AST level was in the traditional low-normal range (10–19 U/L), regardless of the FLI. Moreover, very high AST (≥ 50U/L) was most associated with high CCI. Similarly, higher GGT was associated with CCI, whereas the reference GGT level was low-normal, regardless of the FLI. These findings are notable in light of the fact that the reference ALT level for CCI was relatively high (30–49 U/L) in the high FLI group. These differences in liver enzymes may be due to the impairments in organs these liver enzymes mainly exist.

### Limitations

First, the cross-sectional design of this study precludes establishing a causal relationship between lowest ALT and high CCI. Second, the reference ALT level observed here may differ in studies conducted in other populations with different age distributions and prevalence in comorbidities including fatty liver. Third, FLI does not always reflect fatty liver. Although we had data on diagnosed fatty liver by attending physicians, the methods used for assessment of fatty liver were unclear, and only a limited number of participants underwent fatty liver evaluations such as ultrasound examination, CT, or MRI. Therefore, further study including abdominal images are needed to warrant current results, albeit FLI reflects obesity- or fatty liver-related metabolic abnormalities [17], which might have more important clinical implications. Future cohort studies are needed to determine whether this noninvasive biomarker—a combination of low ALT and fatty liver—can be useful for identifying at-risk patients and promoting public health.

### Conclusions

This study demonstrated a robust association between low ALT and high CCI among individuals with a high FLI, suggesting that the uncommon coexistence of low ALT and fatty liver may reflect older women with low physical activity, and severe comorbidity, which might eventually link to long-term elevated mortality risk. These findings further suggest that the optimal ALT level associated with such conditions might be higher than previously considered and may reflect a phenomenon related to the obesity paradox. Further studies, preferably long-term cohort study, are needed to confirm current results and to determine the optimal ALT level for comorbidities.

## Data Availability

Any inquiries regarding supporting data availability of this study should be directed to the corresponding author.
